# The General Practitioner as Anæsthetist

**Published:** 1910-03-05

**Authors:** J. Allan


### THE GENERAL PRACTITIONER'S COLUMN.

[Contributions to this Column are invited, and, if accepted, will be paid for.]

### THE GENERAL PRACTITIONER AS ANÆSTHETIST.

By J. ALLAN, M.D.

The administration of anæsthetics is in some quarters looked upon as work for a specialist, but there is no reason why it should not be legitimately done by the general practitioner. The duty of giving an anæsthetic cannot, however, be lightly undertaken, and the public have a right to demand that those who give anæsthetics should have had adequate training in this branch of medicine.

### Choice of an Anæsthetic.

For major operative work, where prolonged anæsthesia is required, the choice naturally rests with chloroform or ether or a mixture of these. For several reasons chloroform has been the choice of the majority of practitioners. Perhaps the chief reasons are that chloroform can be so easily carried about and that it can be so easily administered. Ether has never been so much favoured by the man in general practice, probably owing to the greater difficulty of transport of the Clover's apparatus and because of the supposed greater trouble in administering it. There is now a general consensus of opinion in favour of the exhibition of ether by the open method, but the difficulty is that large quantities are used, the mask becomes saturated, and, unless great care is exercised, the ether may get on to the patient's face. This last risk may to a certain extent be obviated by employing three or four layers of lint or flannel in the construction of the mask and by interposing between the face and the edge of the mask a layer of wool or gauze. A mixture of chloroform and ether in the proportion of one of chloroform to two of ether ($C_1 E_2$), or two of chloroform to three of ether ($C_2 E_3$), is an excellent and efficient anæsthetic and is certainly much safer than pure chloroform. As a routine general anæsthetic this mixture is about as good as any. As regards fatality, ether is undoubtedly less dangerous than chloroform, but it is more disagreeable to take and the after effects are likely to be more marked. It is more liable to be followed by pulmonary complications and its exhibition is contra-indicated in patients suffering from bronchitis, etc. For this reason it is not an ideal anæsthetic for old people who are the subjects of chronic bronchitis. It may be incidentally mentioned that many of these old people take and bear chloroform very well indeed. Ether, when administered with freer admixture with air, is not so liable to cause severe after effects or to be followed by lung complications. For minor surgical work ethyl chloride can be used, but it is only suitable for operations which can be accomplished in a few minutes. As a preliminary anæsthetising agent, prior to the exhibition of chloroform or ether, it is excellent. It must not, however, be recklessly administered, because as regards fatality it is intermediate between chloroform and ether, and it therefore cannot be called a safe anæsthetic. It would appear that it cannot be given without risk to a patient in the sitting posture, for example, for teeth extractions in a dentist's chair. Nitrous oxide is the best anæsthetic to use for minor dental work, and it can also be employed in simple surgical operations as in opening abscesses, incising whitlows, etc., where anæsthesia of short duration is only necessary.

### Anæsthetist's Armamentarium.

There are certain things which should always be at hand when an anæsthetic is given, no matter how simple the case may be. These necessary items are tongue forceps, mouth gag, hypodermic syringe charged with strychnine or other cardiac stimulant, and perhaps a mask. Of course, the anæsthetic might be given by means of a towel or piece of lint, but my own experience is that it can be more easily and more satisfactorily given on a mask. No anæsthetic case should be undertaken unless a hypodermic syringe in working order and charged with strychnine is at hand. It takes time to fill a hypodermic syringe, and the prompt injection of strychnine may avert a fatal syncope. It is always a wise precaution to be prepared for emergencies. While the things just mentioned may be taken as the minimum with which an anæsthetist should work, he will prefer to have a better stocked table beside him. In addition to the above, he will probably desire one or two towels, a basin, an extra hypodermic syringe, a minim measure-glass, amyl nitrite capsules, brandy, solution of strong ammonia, throat sponges, etc.

### Some Practical Points.

The patient should be properly prepared; that is to say, the bowels should have been thoroughly moved and no food taken for some hours prior to the exhibition of the anæsthetic. These precautions diminish the risk of accidents during anæsthetisation and tend to minimise after-effects. The condition of the various organs, heart, lungs, kidneys, should always be previously carefully investigated. For instance, a child suffering from several boils is put under a general anæsthetic to allow of these being treated surgically. The child afterwards sinks into a fatal coma, and it is then discovered too late that there is sugar in the urine. The anæsthetic should be given slowly and continuously by constantly dropping it on to the mask from a special bottle. The mask should not be saturated with the anæsthetic and then placed tightly down on the patient's face. Once surgical anæsthesia is established this condition is best maintained by frequently dropping a little of the anæsthetic on to the mask. When struggling, the patient should not be forcibly held down. It is better to simply guide the patient's movements so that he does not harm himself or other people. During this stage the anæsthetic should be given well diluted. Nothing is more calculated to risk the patient taking an overdose than the holding of the mask saturated with the anæsthetic close to the patient's face during the time he is struggling. Speaking generally, one may say that surgical anæsthesia is indicated by muscular relaxation, regular breathing, contracted pupils and absence of corneal reflex. All four may not be present, and it is quite possible to have perfect anæsthesia without the abolition of the corneal reflex. In conclusion, it may be said that the practitioner who undertakes to give the anæsthetic for a major operation should realise that his duty is to attend to the administration of the anæsthetic and to watch carefully the effect on the patient.

